# Minimally Invasive Surgery in Port Harcourt, Nigeria: Progress So Far

**DOI:** 10.7759/cureus.32049

**Published:** 2022-11-30

**Authors:** Rex F Ijah, Emeka Ray-Offor, Patrick O Igwe, Onyeanunam N Ekeke, Philemon E Okoro, Tamunomie K Nyengidiki, Jack O Omodu, Vaduneme K Oriji, Emmanuel O Ocheli, Jude E Okohue, Nze Jebbin, John I Ikimalo

**Affiliations:** 1 Department of Surgery, Rivers State University Teaching Hospital, Port Harcourt, NGA; 2 Department of Colorectal Surgery, Ellen Leifer Shulman and Steven Shulman Digestive Disease Institute, Cleveland Clinic Florida, Weston, USA; 3 Department of Surgery, University of Port Harcourt Teaching Hospital, Port Harcourt, NGA; 4 Department of Surgery, University of Port Harcourt, Choba, NGA; 5 Department of Obstetrics and Gynaecology, University of Port Harcourt Teaching Hospital, Port Harcourt, NGA; 6 Department of Obstetrics and Gynaecology, Madonna University, Port Harcourt, NGA

**Keywords:** minimally invasive surgery, nigeria, port harcourt, cost, audit

## Abstract

Background

The global practice of minimally invasive surgery (MIS) has progressed from basic to advanced procedures. Consequent to this, almost all surgical procedures can be performed through a minimally invasive technique. This study aims to audit the practice of MIS in healthcare facilities within a city in a developing country in Africa.

Methods

This is a multicenter, multispecialty, retrospective descriptive study of minimally invasive diagnostic and therapeutic surgeries performed in private and public health care facilities in Port Harcourt, Rivers State, Nigeria, conducted for a duration of 10 years, from January 2010 to December 2019. A proforma was distributed for completion to identified surgeons from the included study centers. Data on MIS, including types of procedures, time trends, frequency, category of surgery, and cost, were collated. Statistical analysis was performed using IBM Statistical Package for the Social Sciences (IBM SPSS version 20.0, New York, USA).

Results

There were 5845 minimally invasive procedures performed during the study period, out of which only 92 (1.57%) were carried out in government-owned hospitals. Of these, 2570 were gynecologic (44.0%), 1873 were urologic (32.0%), 1300 were general surgeries (22.2%), 142 were pediatric surgeries (2.4%), and 3 (0.05%) were thoracic minimally invasive procedures performed within the 10-year period. The cost of procedures ranged from <$200 USD to >$2000 USD. The hospital stays ranged from <1 day to a maximum of 13 days.

Conclusion

The practice of MIS has made significant progress but has been primarily driven by the private sector. Subsidizing the cost of MIS procedures in government-owned hospitals is likely to improve patronage and improve the skills of surgeons.

## Introduction

Minimally invasive surgery (MIS) is defined as the achievement of therapeutic and diagnostic surgical objectives with the least amount of stimulus so that metabolic, cardiorespiratory, and physiologic effects are minimized [1]. The use of small incisions to achieve minimal invasiveness is considered minimal access surgery [1]. Though in practice, these two expressions are used interchangeably, in this study, the term minimally invasive surgery (MIS) is preferred. The global practice of MIS has progressed from basic to advanced procedures, such that almost all surgical procedures can be performed through minimally invasive techniques [2-8]. Most surgical subspecialties have evolved ways of harnessing the benefits of minimally invasive techniques, which include reduced postoperative pain, a short hospital stay, and an early return to work.

Broadly, there are six domains of minimally invasive surgery comprising endo-luminal surgery, laparoscopic surgery, thoracoscopic surgery, peri-visceral surgery, intra-articular (arthroscopy), and combined approaches [1]. There are many events of success noted in pediatric surgery, thoracoscopic surgery, arthroscopic and spinal surgery, gynecologic surgery, urologic surgery, and general surgery [2-8]. The learning curve of individual surgeons is directly related to the frequency of surgeries performed [9]. Evidently, the performance of a new procedure tends to improve with experience [10]. Hence, the frequency of performing minimally invasive surgery, i.e., the volume of cases, is relevant to the assessment of the progress and success of this surgical practice [11]. An improvement in the performance of laparoscopic surgery leads to a reduction in the operating time and conversion rate with a rise in the frequency of performing particular surgeries [12].

A few surgeon-based or center-based MIS study reports are available in Port Harcourt, Nigeria, but the actual volume of work done is largely under-reported and unverified [13-19]. There are many unanswered research questions on minimally invasive surgery practice. In a low- and middle-income country (LMIC) where challenges abound that mitigate a technology-based surgical practice, it is reasonable to adopt a city-based reporting approach for robust data collection [20-23]. The extent of progress over the early years of practice can be glimpsed from a city-based registry. This study aims to audit the practice of MIS by specialist surgeons in the city of Port Harcourt, Nigeria.

## Materials and methods

Study design

A multi-center, cross-sectional descriptive observational study of all diagnostic and therapeutic MIS procedures performed by surgeons in a metropolis of Nigeria.

Study setting

This multi-specialty study was an audit of all MIS procedures performed by trained Nigerian surgeons in private and public healthcare facilities within the Port Harcourt metropolis conducted for a duration of 10 years, from January 2010 to December 2019. Port Harcourt is the cosmopolitan capital city of Rivers State, Nigeria, which has a land mass of 1811.6 km^2^ area, and a population of about 1.5 million [24,25]. It is a port city in a crude oil-producing state of Southern Nigeria with local and foreign oil industry workers.

Participants

The participating centers and surgeons were identified from reports of the International College of Surgeons Conference Workshop on Minimal Access Surgery 2016, which was held in Port Harcourt and from a facility survey. Ethical clearance was obtained from each health facility with de-identified data collection. A study proforma was shared with identified lead surgeons in each of the included facilities for completion. Identified surgeons who had relocated to another state or declined consent for participation were excluded.

Variables

Data on the type of MIS procedure performed in the different specialties, the total number of cases done, the year duration of the procedure, hospital stays, and the cost of the procedure were imputed into Microsoft Excel (Microsoft Corporation, Redmond, Washington, USA) spreadsheets. Validation of the data with center records was done by the lead author.

Subgroup categorization 

The duration of the MIS procedure was subcategorized into five groups: I = <30 min; II = 30-59 min; III = 60-120 min; and IV = >120 min. The hospital stays were categorized as day-case for walk-in patients undergoing MIS procedures and discharged home on the same day or back to the referral facility. An overnight stay was recorded for patients admitted on the day of MIS for the procedure and remained in the MIS facility for follow-up for the next 24 h. The admissions of walk-in patients post-procedure lasting for <72 h were recorded as short stays. Lastly, patients who were admitted prior to or on the day of the MIS procedure and remained hospitalized >72 h post-procedure were recorded as in-patients. The cost of each procedure was estimated in US dollars at a conversion rate of three hundred and fifty nairas (N 350) to one dollar ($1 USD), the prevailing rate at the time of the study.

Statistical methods

Statistical analysis was performed using IBM Statistical Package for the Social Sciences (IBM SPSS version 20.0, New York, USA). Continuous variables were reported as means and standard deviations, and categorical variables as simple proportions and percentages.

## Results

Study characteristics

**Figure 1:**
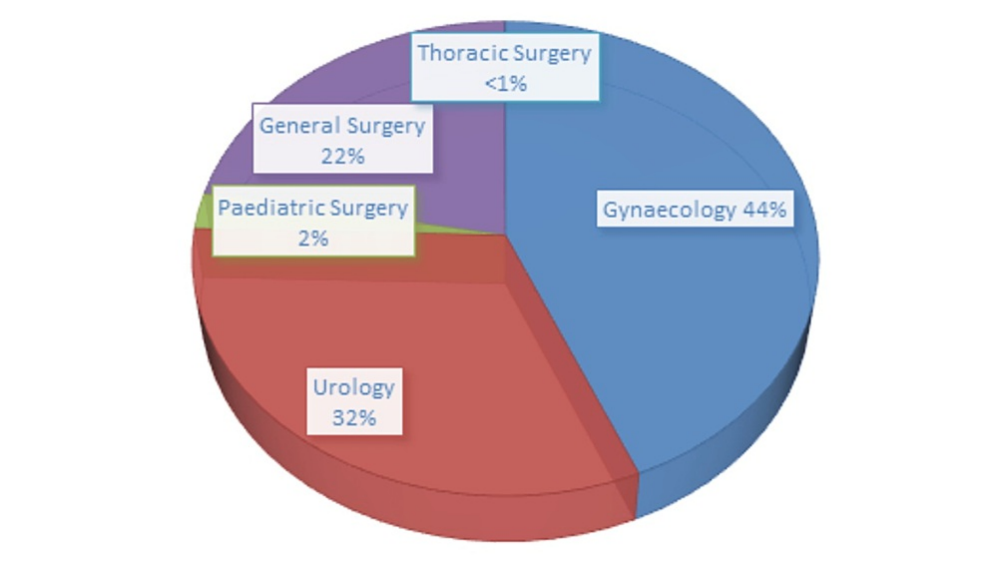
Specialty distribution of MIS procedures during the study period. MIS: minimally invasive surgery.

Thirteen centers that perform minimally invasive surgeries were included in this study; out of these, 12 were privately owned hospitals. In all, 5845 MIS procedures were performed within the study period. Of these, only 92 (1.66%) procedures were performed in the public sector (government-owned hospitals). According to specialties, there were 2570 (44%) gynecologic, 1873 (32%) urologic, 1300 (22%) general surgery, 142 (2%) pediatric surgery, and 3 (0.03%) thoracic surgery procedures performed during the period of study (Figure 1).

Time trend of MIS

**Figure 2:**
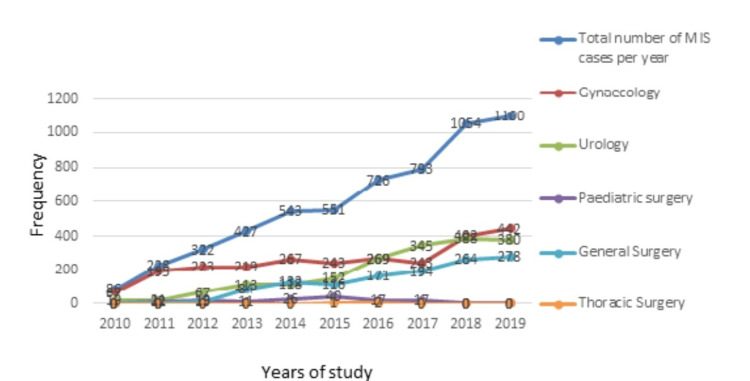
Time trend of MIS procedures performed across subspecialties during the study period. MIS: minimally invasive surgery.

A stepwise rise in the volume of cases was noted in the majority of surgery specialties (Figure 2).

Gynecology

In the first year of the study (2010), 67 gynecologic MIS procedures were performed, comprising diagnostic hysteroscopy-56, hysteroscopic adhesiolysis-5, hysteroscopic polypectomy-2, and a sole case of laparoscopic ovarian cystectomy (Table 1).

**Table 1:** Year-based gynecologic MIS procedures performed during the study period. MIS: minimally invasive surgery.

Procedure	Year										
	2010	2011	2012	2013	2014	2015	2016	2017	2018	2019	Total (%)
Diagnostic hysteroscopy	56	180	205	199	230	221	234	207	332	310	2174 (84.6)
Hysteroscopic adhesiolysis	5	8	3	3	14	10	15	15	38	82	193 (7.5)
Hysteroscopic polypectomy	2	1	1	1	0	0	0	0	0	5	10 (0.4)
Hysteroscopic myomectomy	0	2	6	6	14	11	9	8	15	21	92 (3.6)
Hysteroscopic removal of missing intra-uterine contraceptive device	0	0	0	0	1	0	0	0	0	3	4 (0.2)
Laparoscopic salpingectomy	0	0	2	5	1	0	0	2	2	2	14 (0.5)
Laparoscopic myomectomy	0	0	0	4	3	0	5	1	3	0	16 (0.6)
Diagnostic laparoscopy	3	3	5	0	2	1	4	3	4	6	31 (11.5)
Laparoscopic ovarian cystectomy	1	1	1	1	2	0	2	7	6	5	26 (1.0)
Laparoscopic dye test	0	0	0	0	0	0	0	0	2	8	10 (0.4)
Total (%)	67 (2.6)	195 (7.6)	223 (8.7)	219 (8.5)	267 (10.4)	243 (9.4)	269 (10.5)	243 (9.5)	402 (15.6)	442 (17.2)	2570 (100)

A steep rise in case volume to 195 cases was recorded in the second year, followed by a steady rise over the following years of study to a total of 2570 cases. Diagnostic hysteroscopy accounted for 84.6% of the total caseload, 2174/5845. 

Urology

In the first year of study, there were 19 urologic MIS procedures (1 transurethral resection of the prostate (TURP), 8 direct vision internal urethrotomies (DVIU), and 10 urethrocystoscopies), and by the year 2019, there were 380 MIS procedures per year (Table 2).

**Table 2:** Year-based urologic MIS procedures performed during the study period. MIS: minimally invasive surgery.

Procedure	Year										
	2010	2011	2012	2013	2014	2015	2016	2017	2018	2019	Total (%)
Transurethral resection of prostate (TURP)	1	0	21	37	31	44	94	66	85	101	480 (25.6)
Channeling TURP	0	0	2	1`	1	2	2	1	3	5	17 (0.9)
Direct vision internal urethrotomy (DVIU)	8	10	13	16	21	18	21	23	11	14	155 (8.3)
Cystolithotripsy	0	0	0	0	1	1	1	2	0	1	6 (0.3)
Removal of double J stent	0	0	0	0	0	0	0	10	21	19	49 (2.6)
Cystoscopy and bladder mass biopsy	0	0	2	5	3	6	5	9	8	10	48 (2.6)
Percutaneous nephrolithotomy (PCNL)	0	0	0	0	2	4	1	3	1	3	14 (0.7)
Laser lithotripsy	0	0	2	3	2	0	0	5	17	10	39 (1.9)
Transurethral resection of bladder tumor (TURBT)	0	0	0	1	3	0	0	0	0	3	7 (0.4)
Urethrolithotripsy	0	0	0	0	1	1	2	3	0	2	9 (0.5)
Urethrocystoscopy	10	11	27	50	53	76	143	223	241	213	1047 (55.9)
Laparoscopic renal cortical cystectomy	0	0	0	0	0	0	0	0	1	1	2 (0.1)
Total (%)	19 (1.0)	21 (1.1)	67 (3.6)	113 (6.0)	118 (6.3)	152 (8.1)	269 (14.4)	345 (18.4)	388 (20.7)	380 (20.3)	1873 (100)

Diagnostic urethrocystoscopy was the most frequently performed procedure in this specialty with a total number of 1047 (55.9%), followed by TURP, 480(25.6%).

General Surgery

Laparoscopic surgery in general surgery was introduced in the year 2011 with three laparoscopic appendectomies; upper gastrointestinal endoscopy was recorded the following year and subsequently, flexible lower gastrointestinal endoscopy became routine practice (Table 3).

**Table 3:** Year-based report of aerodigestive endoscopy and laparoscopic general surgery procedures performed during the study period.

Procedure	Year										
	2010	2011	2012	2013	2014	2015	2016	2017	2018	2019	Total (%)
Laparoscopic surgery											
Diagnostic laparoscopy	0	0	3	13	8	1	4	8	5	5	47 (3.6)
Laparoscopic appendicectomy	0	3	3	2	0	0	0	2	1	2	13 (1.0)
Laparoscopic-assisted orchidectomy	0	0	1	0	0	0	0	0	0	1	2 (0.2)
Laparoscopic cholecystectomy	0	0	0	5	1	1	0	0	2	4	13 (1.0)
Laparoscopic adhesiolysis	0	0	0	2	0	0	0	1	0	0	4 (0.3)
Laparoscopic varicocelectomy	0	0	0	0	1	0	0	0	0	0	1 (0.07)
Laparoscopic herniotomy	0	0	0	1	1	0	0	0	0	0	2 (0.2)
Laparoscopic liver biopsy	0	0	0	0	0	0	0	0	1	0	1 (0.07)
Laparoscopic liver cyst aspiration	0	0	0	0	0	0	0	1	0	1	2 (0.2)
Laparoscopic fundoplication	0	0	0	0	0	0	0	0	0	1	1 (0.07)
Laparoscopic foreign body retrieval	0	0	0	0	0	0	0	0	0	1	1 (0.07)
Sub-total	0	3	7	23	11	2	4	12	9	11	87 (6.7)
Aerodigestive endoscopy											
Esophagogastroduodenoscopy	0	0	8	44	61	52	77	68	73	124	507 (39.0)
Colonoscopy	0	0	0	13	42	43	66	67	151	195	487 (37.5)
Sigmoidoscopy	0	0	0	3	7	13	8	14	3	4	52 (4.0)
Diagnostic proctoscopy	0	0	1	1	0	0	0	0	0	0	2 (0.2)
Therapeutic proctoscopy	0	0	0	0	11	5	12	19	5	3	55 (4.2)
Follow-up proctoscopy	0	0	0	0	0	1	4	3	5	5	18 (9.1)
Proctosigmoidoscopy	0	0	0	0	0	0	0	0	4	4	8 (0.6)
Endoscopic retrograde cholangiopancreatography	0	0	0	0	0	0	0	0	0	2	2 (0.2)
Bronchoscopy	0	0	0	0	0	0	0	0	0	2	2 (0.2)
Laryngoscopy	0	0	0	0	0	0	0	1	0	0	1 (0.07)
Subtotal (%)	0	0	9	61	121	114	167	172	241	339	1219 (93.8)
Total (%)	0 (0)	0 (0)	13 (1.0)	84 (6.5)	132 (10.2)	116 (8.9)	171 (13.2)	184 (14.2)	250 (19.2)	350 (27.7)	1300 (100)

The most frequently performed MIS procedure in this specialty was esophagogastroduodenoscopy, 507 (39.0%), followed by colonoscopy, 487(37.5%). In all, 87(6.7%) laparoscopic surgeries were recorded, and half of these were diagnostic laparoscopies. The two most common therapeutic laparoscopic procedures were laparoscopic appendicectomy and cholecystectomy in equal numbers, 26 (30%).

Pediatric Surgery

Pediatric MIS was introduced in 2011 with 12 procedures and has progressed to the year 2017. The highest number of procedures (40 cases) was recorded in 2015 (Table 4).

**Table 4:** Year-based pediatric MIS procedures performed during the study period. MIS: minimally invasive surgery.

Procedure	Year										
	2010	2011	2012	2013	2014	2015	2016	2017	2018	2019	Total (%)
Transurethral ablation of posterior urethral valve	0	2	0	0	0	0	0	0	0	0	2 (1.4)
Diagnostic laparoscopy	0	4	6	5	7	8	1	0	0	0	31 (21.8)
Lap-assisted orchidopexy	0	5	8	4	5	1	4	4	0	0	31 (21.8)
Lap appendectomy	0	1	2	0	0	0	0	0	0	0	3 (2.1)
Sub-total	0	12	16	9	12	9	5	4	0	0	67 (47.2)
Pediatric endoscopy											
Diagnostic upper gastrointestinal endoscopy	0	0	3	0	6	13	5	6	0	0	33 (23.2)
Diagnostic lower gastrointestinal endoscopy	0	0	0	0	7	13	5	6	0	0	31 (21.8)
Therapeutic lower gastrointestinal endoscopy	0	0	0	0	0	2	0	1	0	0	3 (2.1)
Urethrocystoscopy	0	0	0	2	1	3	2	0	0	0	8 (5.6)
Sub-total	0	0	3	2	14	31	12	13	0	0	75 (52.8)
Total (%)	0 (0)	12 (8.4)	19 (13.4)	11 (7.7)	26 (18.3)	40 (28.2)	17 (12.0)	17 (12.0)	0 (0)	0 (0)	142 (100)

The most frequently performed MIS procedure was laparoscopy 65(45.7%), and half of the cases were diagnostic.

Thoracic Surgery

There were three cases of video-assisted thoracoscopies (VATS) performed during the study period, as shown in Table 5.

**Table 5:** Year-based thoracic MIS procedures performed during the study period. MIS: minimally invasive surgery.

Procedure	Year										
	2010	2011	2012	2013	2014	2015	2016	2017	2018	2019	Total (%)
Video-assisted thoracoscopy surgery (diagnostic)	0	0	0	0	0	1	1	0	0	0	2 (66.7)
Video-assisted thoracoscopic surgery (therapeutic)	0	0	0	0	0	0	1	0	0	0	1 (33.3)
Total (%)	0 (0)	0 (0)	0 (0)	0 (0)	0 (0)	1 (33.3)	2 (66.7)	0 (0)	0 (0)	0 (0)	3 (100)

Cost of procedures

The cost range for gynecologic procedures was from $428.6 USD to $2149.9 USD (Table 6).

**Table 6:** Duration, hospital stays, and cost of gynecologic, pediatric, and urologic surgeries in the study.

Gynecologic procedure	Duration category	Hospital stay	Estimated cost ($ USD)
Diagnostic hysteroscopy	I	Day case	428.6
Hysteroscopic adhesiolysis	II	Day case	528.6
Hysteroscopic polypectomy	II	Overnight	1014.3
Hysteroscopic myomectomy	II	Overnight	1213.3
Hysteroscopic removal of intrauterine contraceptive device	II	Day case	428.6
Laparoscopic salpingectomy	II	Overnight	2128.6
Laparoscopic myomectomy	IV	Overnight	2142.9
Diagnostic laparoscopy	II	Day case	1000.0
Laparoscopic ovarian cystectomy	II	Overnight	2452.9
Laparoscopy and dye test	II	Overnight	1142.9
Urology procedure	Duration category	Hospital stay	Estimated cost ($ USD)
Transurethral resection of prostate (TURP)	III	Short stay	2000.0
Channel TURP	II	Short stay	2142.9
Direct vision internal urethrotomy (DVIU)	II	Short stay	1142.9
Cystolithotripsy	II	Short stay	1142.9
Removal of double J stent	I	Day case	1142.9
Cystoscopy and bladder mass biopsy	I	Overnight	1142.9
Percutaneous nephrolithotomy (PCNL)	II	Overnight	2142.9
Laser lithotripsy	II	Short stay	2142.9
Transurethral resection of bladder tumor (TURBT)	II	Short stay	2142.9
Urethrolithotripsy	II	Short stay	2142.9
Urethrocystoscopy	I	Day case	442.9
Laparoscopic renal cortical cystectomy	III	Overnight	1714.3
Pediatric surgery procedure	Duration category	Hospital stay	Estimated cost ($ USD)
Pediatric urethrocystoscopy	I	Overnight	428.6
Transurethral ablation of posterior urethral valve	I	Overnight	457.1
Pediatric diagnostic laparoscopy	II	Overnight	428.6
Lap-assisted orchidopexy	III	Short stay	514.3
Pediatric laparoscopic appendectomy	III	Short stay	571.4
Pediatric diagnostic upper gastrointestinal endoscopy	I	Day case	171.4
Pediatric diagnostic lower gastrointestinal endoscopy	II	Day case	228.6
Therapeutic lower gastrointestinal endoscopy	II	Day case	428.6

For urologic procedures, the cost range was from $442.9 USD to $2142.9 USD, while that of pediatric MIS procedures was from $171.4 USD to $571.4 USD. General surgery MIS procedures ranged from $142.9 USD to $2786 USD, and thoracic surgical procedures were $285.7 USD and $342.8 USD (Table 7).

**Table 7:** Duration, hospital stays, and cost for general and thoracic MIS procedures performed during the study. MIS: minimally invasive surgery.

Laparoscopic procedures	Duration category	Hospital stay	Estimated cost ($ USD)
Diagnostic laparoscopy	II	Day case	1000
Laparoscopic appendectomy	III	Short stay	1572
Lap-assisted orchidectomy	III	Overnight	1000
Lap cholecystectomy	III	Short stay	2786
Lap adhesiolysis	II	Overnight	1000
Lap varicocelectomy	II	Overnight	1000
Lap herniotomy	III	Overnight	1000
Lap liver biopsy	II	Day case	1000
Lap liver cyst aspiration	II	Day case	1000
Laparoscopic orchidectomy	II	Day case	2000
Laparoscopic foreign body retrieval	I	Day case	429
Aerodigestive endoscopy	Duration category	Hospital stay	Estimated cost ($ USD)
Diagnostic esophago-gastro-duodenoscopy	I	Day case	171.4
Therapeutic esophago-gastro-duodenoscopy	II	Day case	571.4
Diagnostic colonoscopy	II	Day case	228.6
Therapeutic colonoscopy	III	Day case	742.9
Diagnostic flexible sigmoidoscopy	I	Day case	157.1
Therapeutic flexible sigmoidoscopy	I	Day case	157.1
Diagnostic proctoscopy	I	Day case	114.3
Therapeutic proctoscopy	I	Day case	171.4
Follow proctoscopy	I	Day case	71.4
Rigid procto-sigmoidoscopy	I	Day case	157.1
Endoscopic retrograde cholangiopancreatography (ERCP)	IV	Day case	714.3
Bronchoscopy	II	Day case	428.5
Laryngoscopy	I	Day case	142.9
Thoracic minimal invasive surgeries	Duration category	Hospital stay	Estimated cost ($ USD)
Video-assisted thoracoscopic surgery (diagnostic)	II	In-patient	285.7
Video-assisted thoracoscopic surgery (therapeutic)	II	In-patient	342.8

Duration of procedures and length of hospital stay

The duration of MIS procedures ranged from <10 min to 3 h. The length of hospital stays was predominantly <24 h (day-case), followed by overnight stays. Thoracic MIS had longer hospital stays (in-patient status) compared to all other specialties.

## Discussion

This city-based report of minimally invasive surgical practice provides a panoramic view of 5845 procedures performed in Port Harcourt, Nigeria, over the early years of this service. It highlights the increasing frequency of gynecologic, urologic, and general surgery procedures performed in this cosmopolitan city. It differs from center-based studies earlier reported in Nigeria describing specialty-specific rates and outcomes of laparoscopic surgeries or endoluminal minimally invasive procedures in the fields of urology, gynecology, or surgical gastroenterology [13,19,26,27]. A cursory look at the total caseload of minimally invasive surgical procedures appears impressive; however, this fades in significance when the caseload per year is considered relative to the city population. The yearly rate of caseload rose during the study period from 67 to 442 in gynecology, 19 to 380 in urology, and 0 to 350 for general surgery, and there was a temporary cessation in growth for pediatric and cardiothoracic surgeries. The progress made in MIS practice during the study period has been significant but slow. These specialties were all operating in the same environment and so the likely explanation for the rate of procedures performed could be patient awareness, availability of equipment, and surgeon factor.

Unpublished historical reports suggest that MIS commenced in the city at a private gynecology center (Prime Medical Consultants) in 2006, followed by a private urology center (Shawsand Medical Center) in 2007 [14]. A private sector predominance in MIS service is observed in this 10-year study, with a staggering 98.3% of the surgical data emanating from these non-government health facilities. This implies that the majority of the less privileged in this low- and middle-income country (LMIC) setting is possibly deprived of the benefits of this modern surgical technique. Also, trainee surgeons and medical students are not exposed to this technology-based practice, which impacts negatively on skill acquisition. A non-comprehensive health insurance coverage in Nigeria and poor funding of the public health sector are other mitigating factors, considering the cost of setup, equipment maintenance, and training of personnel needed for an efficient MIS practice. In climes with efficient social service delivery like Norway, public (government-funded) hospitals record higher numbers of surgical procedures than private health facilities [28].

Overall, gynecologic procedures recorded more case volume than the other specialties, with the most common procedure being hysteroscopy, 2473 (42.3%). Endo-laparoscopic gynecologic procedures performed in the early years of this practice within the city were an average of 228 per year. A possible explanation for this comparative precedence could be more personnel, shorter learning curve, cost of equipment, consumables, and procedure. For endo-urology, an average of 187 adult procedures per year were recorded. Diagnostic urethro-cystoscopy was the most frequently performed procedure in this specialty, 1047 (17.9%), followed by the therapeutic procedure of TURP, 480 (8.2%). There are some center-based study reports of endo-urology services from the city that highlight the applications of endo-urology procedures [13,14,16].

General surgery-related MIS procedures were the third most frequent in this study, 1300 (22.2%), with an average frequency of 144.4 per year. Endoluminal gastrointestinal procedures were more frequently performed than laparoscopic procedures. The spectrum of the gastrointestinal disease diagnosed afforded a vast array of therapeutic endoscopic treatment options [18,29]. A total of 87 laparoscopic general surgery procedures were recorded, with laparoscopic appendicectomy and cholecystectomy as the two most frequent therapeutic procedures. A pilot study on laparoscopic surgeries performed in the city (2012) reported on a smaller study population comprising diagnostic and therapeutic procedures [15]. Studies on laparoscopic surgery in Nigeria similarly report these two therapeutic laparoscopic surgeries as the most frequently performed [26,27]. In a pilot study from another LMIC African setting, laparoscopic appendicectomy and cholecystectomy were similarly reported as the most frequent therapeutic laparoscopic surgeries [12]. One hundred and forty-eight pediatric minimally invasive procedures were performed with an average frequency of 20.2 per year, and a pilot series of three video-assisted thoracoscopic surgeries were recorded. The modest figures from these two specialties were attributed to equipment challenges and the non-ready availability of local maintenance personnel.

In all, the cost of procedures ranged from <$200 to >$2000. There was a marked disparity in the cost of MIS procedures performed in the public sector when compared to the same in private health facilities. A day-case diagnostic laparoscopy in general surgery, for example, costs less than $500 in government-owned health facilities but more than $1000 in private health facilities. It is likely that if the cost is reduced patronage may improve, and the total number of these cases performed may significantly increase. The cost of procedures could have been a deterring factor relative to the open surgical option in an environment where over 70% of people live below the poverty line of less than $2 per day. Although sometimes the cost of procedures may reflect a drive for returns on investment for profit-oriented private sector input, there is a cost-saving advantage when compared with the same treatment outside the country. It may be viewed as a significant private sector contribution to minimizing capital flight from medical tourism [30]. It is observed that the cost of pediatric surgical procedures was comparatively lower than other specialties, ranging from $171.4 to $571.4, yet the total number of cases was low. This implies that there are other factors affecting practice and patronage beyond cost and the art of surgery, like awareness.

Limitations

An approximation of the average duration of surgery, hospital stays, and cost of surgery from all the centers may not reflect accurate information on individual surgeon outcomes. Another limitation is the exclusion of centers that declined consent or relocated to another city. This suggests that the data may be larger than reported. Also, this is a retrospective study from multiple centers and is subject to incomplete data.

## Conclusions

Minimally invasive surgery is a technology-based technique that has modernized surgical practice. The practice of MIS in Port Harcourt, Nigeria, has made significant progress and is primarily driven by the private sector. This study presents the wide spectrum of minimally invasive procedures that were performed in the city during the early years of this practice. The frequency of cases done per year was low, and the average cost of a procedure varied across specialties.

More frequent practice of MIS in public tertiary health facilities is desirable for trainee surgeons to shorten their learning curve. The availability of equipment and instruments for this technology-driven health service requires improved funding, procurement and maintenance of this equipment and training of personnel by the government and private hospitals. Subsidizing the cost of MIS procedures in government and public hospitals is likely to improve patronage and improve the skills of surgeons.
